# Parental Activation and Obesity-Related Health Behaviors Among a Racially and Ethnically Diverse Population of Low-Income Pediatric Patients: Protocol for a Cross-Sectional Survey Study

**DOI:** 10.2196/resprot.9688

**Published:** 2018-11-05

**Authors:** Nakiya N Showell, Corinna Koebnick, Lisa R DeCamp, Margo Sidell, Tatiahna Rivera Rodriguez, Jennifer J Jimenez, Deborah Young, Rachel LJ Thornton

**Affiliations:** 1 Johns Hopkins School of Medicine Division of General Pediatrics and Adolescent Medicine Baltimore, MD United States; 2 Kaiser Permanente Southern California Department of Research and Evaluation Pasadena, CA United States

**Keywords:** activation, parent, child, health behaviors, obesity, primary care

## Abstract

**Background:**

Despite a recent decline in the obesity prevalence among preschool-aged children, obesity remains disproportionately high among children from low-income racial or ethnic minority families. Promoting healthy lifestyles (eg, obesity-preventative behaviors) in primary care settings is particularly important for young children, given the frequency of preventative health visits and parent-provider interactions. Higher adoption of specific health behaviors is correlated with increased patient activation (ie, skill, confidence, and knowledge to manage their health care) among adults. However, no published study, to date, has examined the relationship between parental activation and obesity-related health behaviors among young children.

**Objective:**

The goal of this study is to measure parental activation in low-income parents of preschoolers in 2 large health systems and to examine the association with diet, screen-time, and physical activity behaviors.

**Methods:**

We will conduct a cross-sectional study of parents of preschool-aged patients (2-5 years) receiving primary care at multiple clinic sites within 2 large health care systems. Study participants, low-income black, Hispanic, and white parents of preschool-aged patients, are being recruited across both health systems to complete orally administered surveys.

**Results:**

Recruitment began in December 2017 and is expected to end in May 2018. A total of 267 low-income parents of preschool-aged children have been enrolled across both clinic sites. We are enrolling an additional 33 parents to reach our goal sample size of 300 across both health systems. The data analysis will be completed in June 2018.

**Conclusions:**

This protocol outlines the first study to fully examine parental activation and its relationship with parent-reported diet, physical activity, and screen-time behaviors among low-income preschool-aged patients. It involves recruitment across 2 geographically distinct areas and resulting from a partnership between researchers at 2 different health systems with multiple clinical sites. This study will provide new knowledge about how parental activation can potentially be incorporated as a strategy to address childhood obesity disparities in primary care settings.

**International Registered Report Identifier (IRRID):**

RR1-10.2196/9688

## Introduction

Racial and ethnic minority preschoolers (aged 2-5 years) are disproportionately affected by obesity and are, consequently, at higher risk for obesity-related health outcomes, including adult obesity, diabetes, and cardiovascular disease [1,2]. Effective obesity prevention and treatment programs in clinical settings are a key component of multisector efforts to halt and reverse the childhood obesity epidemic [3]. The American Academy of Pediatrics recommends that obesity screening and healthy weight counseling be integrated into all pediatric well-child visits beginning in infancy and that the counseling include promotion of a healthful diet and developmentally appropriate physical activity behaviors [4]. For young children, this includes paying particular attention to assessing feeding, screen-time, and physical activity-related behaviors and providing anticipatory guidance and counseling [4].

A healthful diet and physical activity behaviors and the adherence to physician recommendations have been linked to patient activation among adults [5,6]. Patient activation refers to skill, confidence, and knowledge in managing one’s health [7]. Research suggests that racial or ethnic minorities have lower activation than white individuals [8]. However, most research in this field focuses on the activation in adults regarding their *own* health.

In spite of the existing evidence base supporting a linkage between the adult patient activation and positive health-related behaviors, research exploring the relationship between parents’ activation on behalf of their children’s health and elucidating the potential mechanisms underscoring these relationships is lacking. Describing the relationship between parental activation and child healthful diet and physical activity behaviors is informative to the development of interventions utilizing parental activation to address disparities in obesity among young primary care patients.

The aims of this project are to examine differences in parental activation by race or ethnicity and explore the relationship between parental activation and parent-reported diet, physical activity, and screen-time behaviors. We hypothesize that the activation will be lower among black and Hispanic parents versus white parents and that parental activation will be positively associated with healthful diet, screen-time, and physical activity behaviors among young primary care patients.

## Methods

### Study Population and Settings

Study participants are being recruited from 2 different health systems, Johns Hopkins Medicine (JHM) and Kaiser Permanente Southern California (KPSC). The JHM pediatric primary care clinics are located in Baltimore, MD, and serve a predominant low-income, black, and Hispanic patient population. Kaiser Permanente, the largest integrated health care system in Southern California, serves a predominantly Hispanic and socioeconomically diverse pediatric population [9,10]. The obesity prevalence and disparities among preschoolers receiving care at KPSC pediatric primary care clinics mirror national trends [11]. About 13.01% (18,315/140,733) of KPSC preschoolers are obese. At this very young age, Hispanic and black preschoolers are more likely to be obese than their non-Hispanic white counterparts (12,703/78,703, 16.14%, and 1340/11,162, 12.01%, respectively, vs 2055/25,967, 7.91%) [11]. At JHM, the pattern for obesity among preschoolers is similar, specifically among young Hispanic patients; 23.3% (211/904) of preschool-aged Hispanic patients are obese compared with 14.1% (35/249) of white patients [12].

### Recruitment Strategies

**Figure 1:**
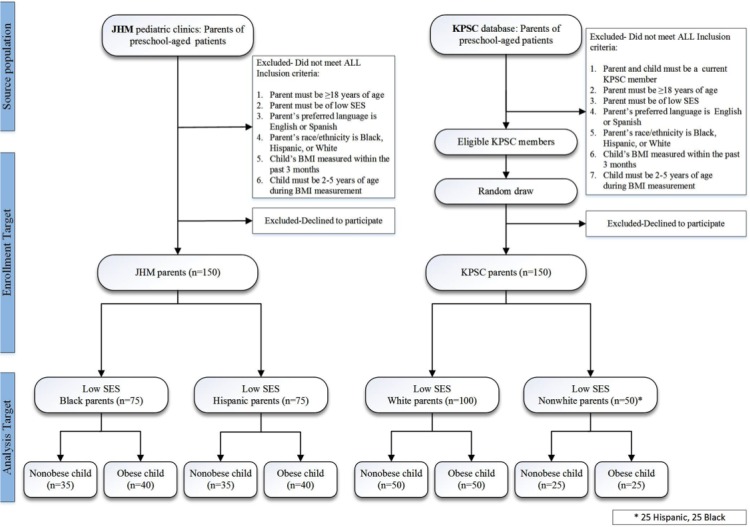
Study design and recruitment targets. JHM: Johns Hopkins Medicine; KPSC: Kaiser Permanente Southern California; SES: socioeconomic status; BMI: body mass index.

Eligible participants are being identified from the electronic medical records (EMRs) at both sites (Figure 1). Parents whose preferred health care language is English or Spanish are eligible to participate if they are (1) aged ≥18 years; (2) of low socioeconomic status (defined by the receipt of Medicaid insurance); (3) a parent of a child aged 2-5 years with height and weight measured in the past 3 months; and (4) black, Hispanic, or white by self-report. From the eligible KPSC source population, lists of potential participants were created in 2 waves by randomly selecting a subset of eligible patients through the EMR. Specifically, an initial random sample of 300 children (200 white and 100 nonwhite) was selected, which consisted of 50% (150/300) nonobese children and 50% (150/300) obese children. The random selection was performed while applying a normal distribution centered at the 50th percentile of body mass index (BMI)-for-age for nonobese and the 97th percentile for obese children. Given the high prevalence of overweight and obesity in this population, the random selection of normally distributed BMIs around the 50th and 97th percentile was chosen to avoid 2 groups of children clustering around the obesity cutoff of the 95th percentile. A second random draw of 175 (100 white, 50 black, and 25 Hispanic children) from the prior sample was then conducted. Oversampling was conducted on the basis of prior response rates to achieve the final intended sample size of 150 children, as indicated in Figure 1. At KPSC, introduction emails were sent to invite randomly selected parents to participate, and follow-up phone calls were conducted to complete surveys. At JHM sites, a convenience sample of low socioeconomic status parents of preschoolers presenting for care is identified based on the EMR review and recruited in real-time during clinic visits to participate in a survey. We will collect data from parents of nonobese (defined as normal weight and overweight: BMI-for-age percentile of 5th-85th) and obese (BMI-for-age percentile ≥95th) children. Because the majority of preschool-aged children will be normal weight, we oversampled parents of children who are overweight or obese. Figure 1 presents the sampling strategy for both clinical sites.

### Data Collection

Surveys are administered in the preferred language of participants (English or Spanish). Surveys will be conducted by telephone at KPSC sites and in-person at JHM sites. The following clinical and sociodemographic data will be collected from children’s EMR: most recent weight, height, BMI or BMI percentile, age, gender, race, ethnicity, and insurance type. The child EMR data are linked with the parental survey data at both sites. Study data are being collected and managed across sites using Research Electronic Data Capture (REDCap) electronic data capture tools hosted at John’s Hopkins Medicine [13]. REDCap is a secure, Web-based app designed to support data capture for research studies, providing (1) an intuitive interface for validated data entry; (2) audit trails for tracking data manipulation and export procedures; (3) automated export procedures for seamless data downloads to common statistical packages; and (4) procedures for importing data from external sources [13]. Table 1 provides a summary of the survey and EMR data currently being collected.

### Measures and Statistical Analysis Plan

Parental activation concerning their child’s health is the primary study outcome and is being assessed using the Parent-Patient Activation Measure (Parent-PAM), a standardized 13-item survey adapted from the well-validated adult Patient Activation Measure (PAM) [7,14]. The Parent-PAM has high internal consistency and reliability among low-income Spanish and English-speaking patients and assesses parents’ knowledge, confidence, and willingness to act concerning their child’s health [14,15]. The Parent-PAM is scored on a Likert scale, and responses are scored on a scale from 0 to 100, with higher scores corresponding to higher activation [14].

Secondary study outcomes are as follows: parent-reported feeding, screen-time, and physical activity behaviors. Parents’ child feeding behaviors will be measured using questionnaires assessing the parental report of feeding sugar-sweetened beverages, fruits and vegetables, and fast food. The survey includes items adapted from the Timing and Frequency of Infant Sugar-Sweetened Beverage Consumption Questionnaire and the Preschool-aged Children’s Physical Activity Questionnaire, which includes measures of screen time [16,17]. Because a prior study found that the parent self-activation was associated with parental activation concerning their child’s health [18], parent self-activation is being measured using the PAM. Furthermore, given the correlation between parent self-activation and activation on behalf of their child’s health, study participants will be randomly assigned to 1 of 2 groups with the alternating ordering of the PAM and related Parent-PAM to minimize order bias in the survey. Multimedia Appendix 1 shows a copy of the survey.

**Table 1:** Collection of electronic medical records and survey data by study site.

Measurement or collection method		Site	
		Johns Hopkins Medicine	Kaiser Permanente Southern California
**Surveys**			
	Parental activation	✓	✓
	Parent self-activation	✓	✓
	Child sociodemographic^a^	✓	✓
	Parent sociodemographic^a^	✓	✓
	Parent feeding, screen-time and physical activity behaviors	✓	✓
	Parent height and weight	✓	✓
	Parent health literacy	✓	✓
	Parent preferred language (medical care)	✓	✓
	Parent English language proficiency	✓	✓
	Parent nativity and immigrant generational status	✓	✓
**Electronic medical records**			
	Child height, weight, or body mass index or body mass index percentile	✓	✓
	Child sociodemographics^a^	✓	✓
	Child medical insurance	✓	✓
	Child health status	✓	✓
	Parent medical insurance^b^	N/A^c^	✓
	Parent body mass index or body mass index percentile^b^	N/A	✓
	Parent sociodemographics^a,b^	N/A	✓

^a^Sociodemographic data collected from surveys and electronic medical records: race and ethnicity, gender, age, educational attainment, employment status, marital status, household income level, neighborhood income, and neighborhood education (determined on the basis of geocoding of addresses and census block information for Kaiser Permanente Southern California site only).

^b^Data not collected from electronic medical records at Johns Hopkins Medicine site.

^c^N/A: not applicable.

Covariates for parents and preschoolers will be included in analyses as follows: (1) parent self-activation (measured using the PAM), parental health care language, health literacy, nativity and immigrant generational status, English language proficiency, race or ethnicity, age, gender, educational attainment, employment status, self-reported height and weight, and study site (JHM or KPSC) and (2) child race or ethnicity, gender, age, health insurance, health status, and BMI.

Descriptive statistics for all variables, including the mean, median, SDs, and frequencies, will be calculated prior to conducting the primary analyses. The Pearson chi-square test or Fisher exact test (in cases of sparse data) for categorical variables and the analysis of variance or the Kruskall-Wallis test for continuous variables will be calculated to assess differences between parental activation scores.

Multivariable regression analyses will be used to test all hypotheses, adjusted for key covariates, including study site. We will consider all covariates as potential candidate variables and will create the final models based on the best fit using the Bayesian information criterion, the corrected Akaike information criterion, and residual analysis. In addition, descriptive analyses will be performed to explore if there are differences by all variables by study site.

To guide our sample size and power estimates for aim 1, we used available data from previously published data in the adult patient activation literature. We estimate a 20% difference in activation levels between racial or ethnic minority and white parents [8]. Assuming a fixed sample size of 300 parents with equal numbers of black, Hispanic, and white parents, we will have 96% power respectively to detect a statistically significant difference (*P*<.05) in parental activation for Hispanic versus white parents.

### Ethics and Consent

The study received approval from the Johns Hopkins’ Institutional Review Board, as well as KPSC’s Institutional Review Board. Informed oral consent is being obtained from all participants. Participants are free to withdraw from the study at any time and can refuse to answer any question.

## Results

Recruitment for the study has commenced at both clinical sites in December 2017. Study enrollment is expected to end by May 2018, and the data analysis is expected to commence in May 2018. Figure 1 provides details on enrollment and recruitment targets.

## Discussion

### Expected Results and Future Directions

Upon completion of this study, we anticipate the following results: parental activation scores for the overall study sample, by clinical site (KPSC vs JHM), and by race or ethnicity (white, Hispanic, and black), noting any detected differences between racial or ethnic subgroups. Additionally, we will have results of analyses testing the association between parental activation and parent-reported healthful diet, physical activity, and screen-time behaviors. Our findings will inform the planning of a larger study. Specifically, if as anticipated, we find a positive relationship between parental activation and obesity-preventative behaviors, we will utilize these findings to guide the development of a primary care-based intervention targeting increasing parental activation to promote healthful diet, screen-time, and physical activity behaviors among low-income preschool-aged children.

### Study Rigor and Reproducibility

To address rigor and reproducibility, a mixed study design is being used—one that incorporates EMR-guided convenience sampling (JHM) and a randomized approach to participant selection (KPSC). The advanced integrated EMR system used by KPSC allows for greater recruitment precision in this study. In addition, we are using the randomization of survey activation measure ordering as a key strategy to minimize survey bias, given the correlation with parent self-activation and parental activation. Such study design approaches improve the level of rigor realistically possible in a study that mimics the real-world clinical recruitment. To promote study reproducibility, we will make every effort to give complete, detailed descriptions of methods and analyses in future publications, given that wide dissemination is a key goal of the project. Details of the recruitment process, participant demographics, and factors for potential response heterogeneity will be published and made available for future replication in other settings.

### Limitations

We acknowledge some limitations of this study. This study uses convenience sampling to recruit participants from the JHM study site. While this sampling strategy is the most practical and feasible recruitment mechanism at this particular site, we recognize that nonrandomized convenience sampling may introduce selection bias; to explore this, we will conduct a comparative analysis of study participants’ characteristics (using demographic and electronic health record data) across the 2 sites. In addition, we will compare study participants to the general population of 2-5-year-old patients at each site to further assess the extent to which study participants are representative of the overall clinic population with respect to demographic characteristics [12]. This comparative analysis will focus on the characteristics that are most likely to be associated with the exposures and outcomes of interests (eg, child BMI distribution, number of clinic visits in a 12-month period).

Furthermore, we will explore if there are any between-site differences in our results, as described in the Methods section. Another limitation of this study is the inability to capture information of other sample characteristics that may be associated with parental activation and obesity-related health behaviors. These factors include the parental perception of the health care system, neighborhood access to healthful food items, and residential proximity to outdoor play space. Future research should explore the influence of additional parent- and neighborhood-level factors on parental activation and its relationship with obesity-related health behaviors.

### Conclusions

This protocol represents the first description of parental activation and its potential association with diet, screen-time, and physical activity behaviors among low-income, racial or ethnically diverse preschool-aged children in primary care settings. Delineation of parental activation among this population and understanding the relationship between parental activation and children’s diet, screen-time, and physical activity behaviors can guide the development of targeted approaches to clinic-based obesity management programs.

## Appendix

Multimedia Appendix 1 Copy of Study Survey.

## Abbreviations

BMI: body mass index
EMR: electronic medical record
JHM: Johns Hopkins Medicine
KPSC: Kaiser Permanente Southern California
PAM: Patient Activation Measure
