# Supplementary material for: Parental Activation and Obesity-Related Health Behaviors Among a Racially and Ethnically Diverse Population of Low-Income Pediatric Patients: Protocol for a Cross-Sectional Survey Study
Source: JMIR Res Protoc. 2018 Nov 5;7(11):e182. doi: 10.2196/resprot.9688 (PMC6246974; doi:10.2196/resprot.9688)
Supplement: Multimedia Appendix 1 [file resprot_v7i11e182_app1.pdf]

KAISER PERMANENTE AND JOHNS HOPKINS MEDICINE RESEARCH COLLABORATION

Assessing the Relationship between Parental Activation and Obesity-related Health Behaviors among Overweight and Obese Low-income Racial/Ethnic Minority Young Patients

Applicants:

Nakiya N. Showell, MD, MPH, MHS (Johns Hopkins Medicine PI)
Assistant Professor of Pediatrics

Deborah Rohm Young, PhD (Kaiser Permanente Southern California PI)
Director, Behavioral Research

Rachel Thornton, MD, PhD (Johns Hopkins Medicine)
Assistant Professor of Pediatrics

Lisa R. DeCamp, MD, MPH (Johns Hopkins Medicine)
Assistant Professor of Pediatrics

Corinna Koebnick, PhD, MSc (Kaiser Permanente Southern California)
Research Scientist II

Claudia Nau, PhD (Kaiser Permanente Southern California)
Research Scientist I

Date: March 10, 2017

Assessing the Relationship between Parental Activation and Obesity-related Health Behaviors among Overweight and Obese Low-income Racial/Ethnic Minority Young Patients

A. Co-applicants: Nakiya Showell, MD, MPH, MHS (JHM PI), Deborah Young, PhD (KPSC PI)
Other Team Members: Rachel Thornton, MD, PhD (JHM), Lisa DeCamp, MD, MSPH (JHM), Corinna Koebnick, PhD, MSc (KPSC), Claudia Nau, PhD (KPSC)

B. Introduction/Rationale: Racial/ethnic minority preschoolers (i.e., 2-5 year olds) are disproportionately affected by obesity and its associated health consequences including adult obesity, hypertension, diabetes and cardiovascular disease (CVD).[1,2] Obesity prevalence and disparities among preschoolers receiving care at Kaiser Permanente Southern California (KPSC) pediatric primary care clinics mirrors national trends. 13.0% of KPSC preschoolers are obese. 16% of Latino and 12% of African American preschoolers are obese compared to 7.9% of non-Hispanic Whites.[3] The pattern for obesity among preschoolers is similar among Johns Hopkins pediatric primary care patients, though the prevalence is generally higher for Latino young patients. 23% of Latino and 14% of African-American patients are obese compared to 14% of white patients.[4]

Effective obesity management programs in clinical settings are a key component of multi-sector efforts to eliminate child obesity disparities and reverse the childhood obesity epidemic. The American Academy of Pediatrics recommends integrating obesity screening and healthy weight counseling into pediatric well child visits starting in infancy.[5] Counseling should promote healthful diet, physical activity (PA), and screen time behaviors. For overweight and obese preschoolers (BMI≥85th percentile), clinicians should also perform in-depth behavioral assessments and targeted counseling addressing sugar sweetened beverage intake, playtime, and feeding practices. This should be paired with continual monitoring of weight trajectories.[5]

Healthful diet and PA behaviors, and adherence to physician recommendations have been linked to patient activation among adults.[6,7] Patient activation refers to skill, confidence and knowledge in managing one's health and is commonly measured using the Patient Activation Measure (PAM); a 13-item scale with strong psychometric properties.[8] Research suggests that lower-income adults and racial/ethnic minority adults have lower activation than higher-income adults and White adults respectively.[9,10] Most research in this field focuses on activation among adult patients regarding their own health. In fact, no studies have described parental activation among racial/ethnically and socioeconomically diverse populations of parents of preschoolers across primary care settings. Furthermore, describing the relationship between parent activation and child diet and PA behaviors is critical to inform development of child obesity interventions in primary care.

The goal of the proposed pilot project is to inform development of future obesity management interventions addressing obesity disparities in preschoolers in primary care settings. It will be achieved through **two Aims:**

Aim 1: Measure activation among a clinical sample of socioeconomically and racial/ethnically diverse parents of overweight and obese preschoolers seeking primary care in two large health systems, and describe the sociodemographic factors associated with parental activation, using a cross-sectional survey design.

Hypothesis 1: Activation levels will be lower for Black and Latino vs. White and low- vs. high-income parents.

Aim 2: Examine the association of parental activation with adherence to healthy feeding, screen time and PA behaviors using a cross-sectional survey design.

Hypothesis 2: Parental activation is positively associated with adherence to healthy feeding, screen time and PA behaviors.

Conceptual Model: The conceptual model for the proposed research (Fig. 1) depicts the influence of sociodemographic factors on parental activation and the hypothesized relationship between parental activation and adherence to healthy feeding, screen time and PA behaviors. Specifically, the proposed research posits that sociodemographic factors predict parental activation and that this activation directly influences healthful feeding, screen time and PA practices.

Figure 1: Conceptual Model

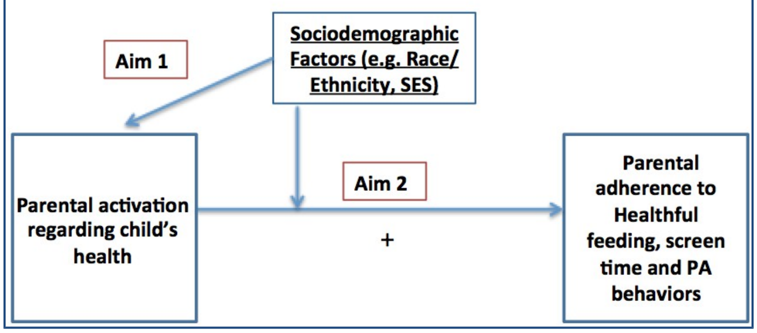

C. Methods: Study participants will be recruited from JHM and KPSC pediatric primary care clinics. Two pediatric primary care clinics in East Baltimore serving low-income, racial/ethnic minority patients at JHM will participate. KPSC serves a racial/ethnic and socioeconomically diverse patient population, with more than 10% of pediatric patients insured through Medicaid.

Study Population and Data Collection: We will recruit a convenience sample of parents of preschoolers presenting for care at the JHM and KPSC sites to participate in a survey. Parents whose preferred healthcare language is English or Spanish will be eligible to participate if they are: 1) ≥ 18 years, 2) the parent of an overweight/obese preschooler (BMI ≥ 85th percentile) with height and weight measured in the past 3 months, 2) White, non-Hispanic Black or Hispanic/Latino by self-report. Preschool patients' EMR data will be linked with parental survey data at both sites. Surveys will be orally administered at JHM. KPSC will identify potential participants through EMR and administer surveys by phone.

Measures: parent self-reported race/ethnicity and parent self-reported socioeconomic status are the independent variables for Aim 1. Parent activation concerning their child's health is the main outcome variable for Aim 1. This will be measured using the Parent Patient Activation Measure (P-PAM), a standardized 13-item survey adapted from the well-validated adult patient activation measure.[11] Parental activation (measured by the P-PAM) is the independent variable for Aim 1. Outcome variables assessed in Aim 2 are: parent self-reported adherence to recommended feeding, screen-time and PA behaviors. Parent's child feeding behaviors will be measured using questionnaires assessing parental report of feeding: sugar-sweetened beverages, fruits and vegetables, and fast food. The survey will also include items adapted from the Child Food Frequency Questionnaire; the Timing and Frequency of Infant SSB Consumption Questionnaire; and the Preschool-aged Children's Physical Activity, which includes measures of screen time.[12-14]

Covariates for parents and preschoolers will be included in analyses: 1) parental healthcare language, English language proficiency, race/ethnicity, age, gender, educational attainment, employment status, and self-reported height and weight*; and 2)* child race/ethnicity, gender, age, health insurance, and BMI percentile.

Analysis Plan: Descriptive statistics will be used to summarize parent activation, parent feeding, screen time and PA behaviors. Multivariate regression analyses will be used to test hypotheses. Specifically, we will determine whether there is a statistically significant difference in parent activation by parent race/ethnicity and socioeconomic status (Aim 1) and determine whether parent activation is associated with parent feeding, screen time, and PA behaviors (Aim 2). These analyses will adjust for key covariates.

Sample Size and Power Calculations: Aim 1: Borrowing from the adult literature, we estimate a 20% difference in activation levels between racial/ethnic minority and white parents, with similar effect size expected for differences in activation level between low- and high-income parents.[9,10] Assuming a fixed sample size of 300 parents with equal numbers of Black, Latino, and White parents; and equal numbers of low- and high-income parents, we will have 83% and 96% power respectively to detect a statistically significant difference ($p<0.05$) in parental activation for Latino vs. White parents and low- vs. high-income parents.

D. Prior Research Experience at Proposed Sites: Drs. Showell, Thornton and DeCamp have a strong track record of collaborative research involving successful recruitment and retention of English and Spanish-speaking low-income parents of preschoolers from the proposed JHM clinics. Drs. Showell and DeCamp have also used the P-PAM with this population. This work has resulted in cultural adaptations of the P-PAM including item modification to address variations in colloquial language among Spanish-speaking parents. Drs. Young and Koebnick have extensive experience using EMR data to link clinical practice and health behavior change among KPSC pediatric patients. They are also highly experienced in participant recruitment.

E. Key Deliverables: Table 1 provides a timeline for achieving all key deliverables during the award period. If funded, this project will produce an NIH or foundation grant proposal to develop a clinic-based childhood obesity intervention, and 2-4 coauthored manuscripts including one comparing parent activation levels among sociodemographic subgroups, and a second describing the association between parent activation and parent feeding, screen time, and physical activity behaviors.

Table 1: Key Deliverables for Pilot Project	Time (months)						
	0	2	4	6	8	10	12
IRB approval	X						
Survey development and refinement	X	X					
Data collection at JHM (2 clinical sites) and KPSC		X	X	X			
Data analysis			X	X	X		
Presentation to Senior Leadership at KPSC						X	
Presentation to Senior Leadership at JHM Pediatrics						X	
Manuscript prep/presentations at national conferences						X	X
Preparation of Collaborative Grant to Develop, Implement and Evaluate Obesity Management Intervention (i.e. NIH R21 or R01)						X	X

References:

1. Ogden CL, Carroll MD, Kit BK, Flegal KM. Prevalence of obesity and trends in body mass index among US children and adolescents,1999-2010. *JAMA*. 2012;307(5):483–490.
2. Wang Y, Beydoun MA. The obesity epidemic in the United States--gender, age, socioeconomic, racial/ethnic, and geographic characteristics: a systematic review and meta-regression analysis. *Epidemiol Rev.* 2007;29:6-28.
3. Koebnick C, Mohan YD, Li X, Young DR. Secular Trends of Overweight and Obesity in Young Southern Californians 2008-2013. *J Pediatr*. 2015 Dec; 167(6): 1264071.
4. Thornton RJ, Showell NN. Living with Violent Crime: Which Neighborhood Factors Influence Obesity Prevalence Among Low-Income Preschoolers? Manuscript in Press.
5. Bright Futures Guidelines for Health Supervision of Infants, Children & Adolescents, Promoting Healthy Weight. Retrieved from: https://brightfutures.aap.org/materials-and-tools/tool-and-resource-kit/Pages/default.aspx. Accessed March 3 2017.
6. Hibbard JH, Greene J. What the evidence shows about patient activation: better health outcomes and care experiences; fewer data on costs. *Health Aff (Millwood).* 2013;32(2):207–14.
7. Greene J, Hibbard JH, Sacks R, Overton V, Parrotta CD. When patient activation levels change, health outcomes and costs change, too. *Health Aff (Millwood).* 2015 Mar 1;34(3):431-7.
8. Hibbard JH, Stockard J, Mahoney ER, Tusler M. Development of the Patient Activation Measure (PAM): conceptualizing and measuring activation in patients and consumers. *Health Serv Res*. 2004 Aug;39(4 Pt 1005-26.
9. Hibbard JH, Cunningham PJ. How Engaged are Consumers in Their Health and Health Care, and Why Does It Matter? Center for Studying Health System Change. 2008 Oct; (8): 1-9.
10. Cunningham PJ, Hibbard J, Gibbons CB. Raising low 'patient activation' rates among Latino immigrants may equal expanded coverage in reducing access disparities. *Health Aff (Millwood)*. 2011 Oct;30(10):1888-94.
11. Insignia Health Parent Patient Activation Measure, http://www.insigniahealth.com. Accessed March 3, 2017.
12. Taveras EM et al. Randomized Controlled Trial to Improve Primary Care to Prevent and Manage Childhood Obesity The High Five for Kids Study. *Arch Pediatr Adolesc Med*. 2011 Aug;165(8):714-22.
13. Benjamin SN et al. [SSB questionnaire] personal communication.
14. Dwyer GM, Hardy LL, Peat JK, Baur LA. The validity and reliability of a home environment preschool-age physical activity questionnaire (Pre-PAQ). *Int J Behav Nutr Phys Act*. 2011 Aug 4;8:86.
